# pH‐Dependent Degradation of Macrobial Environmental DNA in Water

**DOI:** 10.1111/1755-0998.70101

**Published:** 2026-01-16

**Authors:** Toshiaki S. Jo

**Affiliations:** ^1^ Japan Society for the Promotion of Science Tokyo Japan; ^2^ Graduate School of Informatics Kyoto University Kyoto Japan

**Keywords:** biomonitoring, degradation, environmental DNA (eDNA), meta‐analysis, pH, systematic review

## Abstract

Environmental DNA (eDNA) analysis has increasingly been used for aquatic biomonitoring, although interpretation of results needs to consider how environmental factors influence the degradation process of eDNA. This review focuses on pH, which has long been considered a key factor in eDNA degradation although its apparent effect on eDNA degradation has varied across studies. Here I present a synthesis of existing research that summarises what is so far known about the pH dependence of eDNA degradation, and a meta‐analysis that demonstrates a nonlinear, upward convex relationship between eDNA decay rates and pH, with modelled eDNA decay rates peaking around pH 8. These results suggest that under slightly alkaline conditions, which are often considered suitable for DNA preservation, eDNA degradation may be accelerated by the promotion of microbial and enzymatic activity. On the other hand, there were substantial inter‐study discrepancies in the dataset, suggesting that the present meta‐analysis could not fully address the complexity of pH‐dependent eDNA degradation. I end this review with an extensive discussion of some possible mechanisms that should be further investigated in order to achieve a more comprehensive understanding of the pH dependence of eDNA degradation. These efforts will help with more accurate predictions of the persistence time and decay rates of eDNA under various environmental conditions, thereby potentially improving our interpretations of eDNA‐based biodiversity surveys.

## 1 Introduction

Environmental DNA (eDNA) is defined as the total pool of DNA (e.g., cells, organelles and extracellular DNA) isolated from environmental samples including water, soil and air (Rodriguez‐Ezpeleta et al. 2021; Nagler et al. 2022). The majority of eDNA is typically derived from microbes (e.g., bacteria) and their extracellular DNA, with a small portion of extra‐organismal DNA from macro‐organisms (e.g., epidermis, mucus, faeces, gametes and plan stem fragments) (Barnes and Turner 2016; Jo, Takao, and Minamoto 2022). The analysis of eDNA enables inferences of species distributions and abundance without the need for capturing or visually observing organisms, allowing for non‐invasive, cost‐effective and sensitive biomonitoring of various taxa and ecosystems (Fediajevaite et al. 2021; Keck et al. 2022). Environmental DNA‐based biomonitoring is broadly categorised into (i) species‐specific detection, often using quantitative real‐time PCR (qPCR), and (ii) comprehensive detection of specific taxonomic groups using metabarcoding (Nicholson et al. 2020; Scriver et al. 2023). These approaches are increasingly used to supplement or in some cases replace other survey methods, although knowledge gaps remain, for example it is not yet completely understood how long it takes for eDNA to degrade in the environment, nor to what extent spatiotemporal resolution represents biological signals (Jo and Minamoto 2021; Lamb et al. 2022). In conditions that promote rapid eDNA degradation, false negatives may make it difficult to conclude whether target species are in fact present at or near the sampling site. Conversely, in conditions that slow the degradation of eDNA, false positives may occur if eDNA was left by taxa that have not recently occurred within or near that site. A refined understanding of the eDNA degradation process is thus essential for accurate interpretations of eDNA signals and the effectiveness of associated conservation and eradication management plans (Burian et al. 2021; Holman et al. 2022).

Multiple environmental factors that may influence eDNA degradation have been investigated, including biotic factors such as the activity of microbes and their extracellular enzymes (Lance et al. 2017; Jo et al. 2019; McKnight et al. 2024), and abiotic factors such as temperature (Strickler et al. 2015; Snyder et al. 2025), water quality (Eichmiller et al. 2016; Collins et al. 2018), and substrate condition (Brandão‐Dias, Tank, et al. 2023; Zhang et al. 2024). pH has long been considered a key factor in eDNA degradation (Barnes et al. 2014; Strickler et al. 2015; Yang et al. 2025), although its apparent effect on eDNA degradation has varied across studies. Given that depurination (loss of purine bases) and hydrolysis of DNA molecules are generally accelerated under low (i.e., acid) pH conditions (Lindahl 1993; Torti et al. 2015), eDNA degradation should be promoted under acidic conditions and it may therefore be expected that eDNA persists longer under neutral to moderately alkaline compared to acidic conditions (Harrison et al. 2019; Scriver et al. 2023). Some empirical studies have supported this expectation (Strickler et al. 2015; Seymour et al. 2018), although other studies found that eDNA degradation can be accelerated under alkaline conditions (Barnes et al. 2014; Zhao et al. 2023) or may be independent of pH levels (Eichmiller et al. 2016; McKnight et al. 2024). These inconsistent findings may partially explain why pH has overall received less attention than water temperature and flow rate when investigating eDNA persistence and degradation (Harrison et al. 2019; Nicholson et al. 2020).

pH may also indirectly influence eDNA degradation through its influence on microbial and enzymatic activities, which lyse cell and organelle membranes and fragment DNA to consume as a nutrient source (Mauvisseau et al. 2022; Beattie et al. 2023). Bacterial functions and metabolisms also depend on environmental conditions, including pH. Most bacterial communities in soils exhibit optimal growth under approximately neutral pH, and their activity generally decreases in more acidic or highly alkaline soils, although some taxa can adapt to extreme pH conditions (Bååth 1996; Wu et al. 2017; Wan et al. 2020). The activity of DNA‐degrading enzymes, such as nucleases secreted by microbes, is also pH‐dependent and is often suppressed as a result of inactivation and denaturation under acidic conditions (e.g., the optimum pH of DNase I ranges from 7 to 8) (Sigman et al. 1993). Therefore, eDNA degradation might occur more rapidly under neutral to moderately alkaline conditions compared to more acidic conditions, with the relationship between eDNA degradation and pH potentially following an upward convex curve with a peak at neutral to moderately high pH levels, rather than a linear relationship.

This study tests the hypothesis of a non‐linear relationship between pH and eDNA degradation through a quantitative meta‐analysis based on existing research to determine whether and how eDNA decay rate depends on pH and other covariates (temperature, water type, and PCR amplicon length; see below). This is followed by a discussion of factors relevant to a more comprehensive understanding of pH‐driven eDNA degradation. This review focuses on macrobial eDNA because of inherent differences between macrobial and microbial eDNA: microbial eDNA includes living cells which are expected to have different patterns of degradation compared to macrobial eDNA in the form of the extracellular (dead) and intracellular (but metabolically inactive) DNA (Torti et al. 2015; Rodriguez‐Ezpeleta et al. 2021; Nagler et al. 2022). The focus is further refined to aqueous eDNA because of the scarcity of studies that measured both eDNA decay rates and pH in terrestrial environments (see below).

## 2 Materials and Methods

The meta‐analysis aims to assess whether the effect of pH—along with additional covariates—on eDNA decay rates is nonlinear. Referring to the Preferred Reporting Items for Systematic Reviews and Meta‐Analyses (PRISMA) statement (Page et al. 2021), a literature search was conducted using Google Scholar (https://scholar.google.com; the final date of the literature search was July 1, 2025). The search terms were ‘environmental DNA’ AND ‘pH’ AND (‘decay’ OR ‘degradation’ OR ‘removal’ OR ‘persist’), which retrieved approximately 17,200 studies. Their titles, abstracts, and main texts were read, and papers that met all of the following criteria were retained: (i) targeting eDNA from macro‐organisms (not from microbes) in water, (ii) published in international peer‐reviewed journals (not preprint), (iii) published as an original article (not review papers, perspectives, etc.), (iv) estimating the rate of eDNA decay (decrease in eDNA concentration per unit of time) or having a dataset from which the decay rate can be estimated, and (v) measuring pH in water samples at the time samples were collected. Regarding criterion (i), terrestrial eDNA (e.g., soils and sediments) was out of the research focus because there was only one study (Sedlmayr and Schenekar 2024) that measured both eDNA decay rates and pH in terrestrial environments. Regarding criterion (iv), a first‐order exponential decay model is typically used to estimate decay rates (*C*_*t*_ = *C*_0_ e^−*kt*^, where *C*_*t*_ is the eDNA concentration at time *t*, *C*_*0*_ is the initial eDNA concentration, and *k* is the decay rate constant) (Jo and Minamoto 2021; Lamb et al. 2022). Some studies estimated the decay rate using a biphasic exponential decay model, which consists of an initial rapid (*k*_1_) followed by slower (*k*_2_) degradation phases (e.g., Eichmiller et al. 2016; McCartin et al. 2022). However, most of the eDNA signals would be lost during an initial rapid degradation process; thus, if a biphasic model was used, only *k*_1_ was extracted.

All the analyses were performed using R version 4.2.2 (R Core Team 2023). Decay rate constants (unified to per hour) were directly extracted from the main text or supplementary file; estimated from supplementary datasets in the supplementary file using the *nlme* package (Pinheiro et al. 2017); or extracted from figures using WebPlotDigitizer Version 4.6 (https://automeris.io/WebPlotDigitizer/). eDNA decay rates based on different experimental conditions within the same study (e.g., different species, abiotic conditions, or marker) were analysed separately. The number of water samples used for estimating each decay rate was also recorded. As covariates, water temperature [°C], water type, and PCR amplicon length [base pair; bp] were extracted from each study, all of which have been considered in previous meta‐analyses for eDNA degradation (Jo and Minamoto 2021; Saito and Doi 2021; Lamb et al. 2022). Water type was classified into three types: artificial (e.g., tap and distilled waters), freshwater (e.g., well, pond and river waters), and marine (e.g., seawater), following Jo and Minamoto (2021). If necessary, the mean temperature was calculated by averaging the temperatures measured at each sampling time point or the maximum and minimum temperatures during the experimental period.

Random‐effect meta‐analyses were run using the *rma.mv* function in the *metafor* package (Viechtbauer 2010) to assess how pH and other covariates were associated with eDNA decay rate. Before the analyses, all the decay rates were subject to the Yeo‐Johnson transformation (Box‐Cox transformation extended to negative values; Riani et al. 2023) to reduce the skewness. Two types of models were constructed, assuming linear (Model 1) and nonlinear (quadratic; Model 2) effects of pH on eDNA degradation. Both models included eDNA decay rate constants as a dependent variable; pH, temperature, water type, amplicon length, and their primary interaction with pH (pH:temperature, pH:water type and pH:amplicon length) as moderator variables; and study groups as a random effect. In Model 2, a quadratic term of pH (i.e., pH^2^) was also included as a moderator variable, assuming a quadratic degradation curve. Variance inflation factors (VIFs) between covariates ranged from 1.00 to 1.41, indicating that the multicollinearity among the variables was negligible. The inverse of the sample size corresponding to each decay rate constant was included in both models as the sampling variances (i.e., it is assumed that the decay rate constant estimated from a larger number of samples would potentially exhibit larger precision). Model selection was performed based on Akaike's Information Criteria (AIC).

Using the models, residual heterogeneity (the degree of consistency; Nakagawa and Santos 2012) across study groups was evaluated by performing Cochran's *Q* test and calculating the *H* ^2^ statistic (Higgins and Thompson 2002). Publication bias (the propensity for positive and statistically significant research to be published compared with negative and non‐significant ones; Haddaway et al. 2020) was also evaluated by visualising a funnel plot and performing a rank correlation test using the *ranktest* function in the *metafor* package. The residual asymmetry is generally attributed to data points with fewer sample sizes (Yates et al. 2019). In this study, by subjectively drawing a transect on the funnel plot and identifying data points characterising the residual asymmetry, data points based on nine or fewer sample sizes were found to be primarily responsible for this asymmetry and disproportionately large sampling variances. Therefore, if a publication bias was detected, sensitivity analysis excluding decay rate constants with nine or fewer sample sizes was conducted in order to assess whether the main conclusions were robust to the removal of potentially highly imprecise estimates.

## 3 Results

**FIGURE 1:**
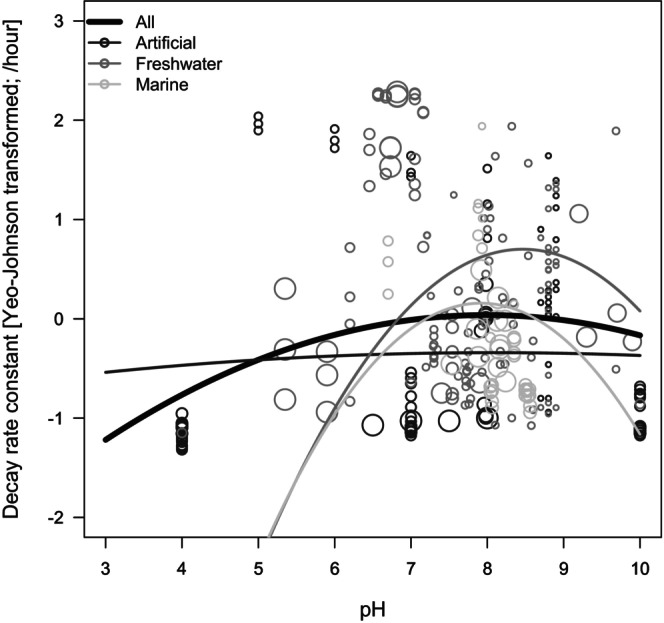
Relationship between eDNA decay rate constant [Yeo–Johnson‐transformed; per hour] and pH (upper left) identified by the meta‐analysis. The size of each plot represents the sample size (the number of water samples required to estimate the decay rate). Regression curves are shown in thick (all data) and thin (dark grey: artificial water, grey: freshwater, light grey: seawater) lines.

In total, 23 studies and 284 decay rate constants (ranging from −0.007 to 1.769 [per hour]) were included in the meta‐analysis (Table S1). Water temperature, pH and PCR amplicon length ranged from 4°C to 35°C, from 4 to 10 and from 73 to 292 bp, respectively. The number of decay rate constants derived from artificial water, freshwater and marine water types was 102, 139 and 43, respectively. Model selection showed that Model 2 (assuming a nonlinear pH effect) had significantly lower AIC than Model 1 (assuming a linear pH effect) (ΔAIC = 47.7; Table 1). In Model 2, both pH and its quadratic term were statistically significant (both *p* < 0.001), and eDNA decay rates were modelled as an upward convex curve with a peak at around pH 8 when using all the datasets (Figure 1). Additionally, water temperature and amplicon length had significant positive effects on eDNA decay rates (*p* < 0.05 and *p* < 0.001). There was no significant difference in the decay rate between artificial water and seawater (*p* > 0.05), while eDNA degradation in freshwater was significantly different from that in artificial water (*p* < 0.001). Moreover, some of the covariates exhibited an interactive effect with pH on the decay rate, inferring that the difference in the decay rate between artificial water and freshwater became narrower and the effect of amplicon length on the decay rate was reversed under higher pH conditions (Table 1; Figure S1). Regardless of the model types, significant residual heterogeneity across datasets (*Q*_*E*_) was detected (*p* < 0.001), which was further supported by *H* ^*2*^ that was substantially larger than 1 (Table 1).

**TABLE 1:** Summary of the random‐effect meta‐analyses.

Variable	Model 1: linear pH effect				Model 2: nonlinear pH effect			
	Estimate	SE	*z* value	95% CI	Estimate	SE	*z* value	95% CI
Moderators								
Intercept	−1.859***	0.488	−3.812	[−2.814, −0.903]	−4.215***	0.582	−7.249	[−5.335, −3.076]
pH	0.287***	0.053	5.373	[0.182, 0.391]	0.991***	0.113	8.799	[0.770, 1.212]
pH^2^					−0.051***	0.007	−7.106	[−0.065, −0.037]
Temperature	0.020*	0.009	2.084	[0.001, 0.038]	0.019*	0.009	2.039	[0.001, 0.038]
Water type (freshwater)	−7.001***	0.390	−17.949	[−7.765, −6.236]	−6.990***	0.389	−17.976	[−7.752, −6.228]
Water type (marine)	−1.251	1.073	−1.166	[−3.354, 0.852]	−1.958	1.074	−1.823	[−4.063, 0.147]
Amplicon length	0.018***	0.003	5.867	[0.012, 0.024]	0.018***	0.003	5.791	[0.012, 0.024]
pH: temperature	−0.001	0.001	−0.673	[−0.003, 0.002]	−0.001	0.001	−0.621	[−0.003, 0.002]
pH: water type (freshwater)	0.805***	0.042	19.385	[0.724, 0.887]	0.805***	0.042	19.402	[0.723, 0.886]
pH: water type (marine)	−0.025	0.150	−0.168	[−0.319, 0.269]	0.077	0.150	0.512	[−0.217, 0.371]
pH: amplicon length	−0.002***	0.000	−5.550	[−0.003, −0.002]	−0.002***	0.000	−5.470	[−0.003, −0.002]
Heterogeneity test								
Residual: *Q*_*E*_	3471.3***				3348.6***			
Moderators: *Q*_*M*_	478.7***				527.5***			
*H* ^2^	12.3				11.8			
Variance components								
$\tau^{2}$	1.507				1.321			
Fit indices								
Log‐like	−638.1				−613.2			
Deviance	1276.2				1226.4			
AIC	1298.2				1250.4			

*Note:* Asterisks indicate statistical significances (***p < 0.001; *p < 0.05). Additional meta‐analyses based on subset data are shown in Table S2.

A rank correlation test and funnel plot indicated the possibility of publication bias in the meta‐analysis (Kendall's tau = 0.22; *p* < 0.001) (Figure S2). As part of the planned sensitivity analysis, the decay rates estimated using nine or fewer samples were removed from the dataset, and the meta‐analysis based on the subset data (resulting in 154 decay rate constants) was re‐run. A subsequent rank correlation test found that the publication bias was not significant (Kendall's tau = 0.03; *p* = 0.65). In the re‐analysis of the subset data, the model assuming a nonlinear pH effect was also significantly supported and the covariates' effects on the decay rate generally exhibited a similar trend as above (Table S2), indicating that the main findings were not driven by small‐sample bias.

## 4 Discussion

### 4.1 Nonlinear Relationship Between pH and eDNA Degradation

By synthesising existing research, this meta‐analysis identified a non‐linearity in eDNA decay rates over a wide pH range. The nonlinear relationship between eDNA degradation and pH was also suggested by a previous synthesis (Mauvisseau et al. 2022), although this was based on a limited number of studies (published before October 2020). The results suggested here, which are based on a larger number of studies, suggest that relative to the physicochemical instability of DNA molecules under acidic conditions, an increase in microbial and enzymatic activities under neutral or slightly alkaline conditions influences the eDNA degradation process. Additionally, this meta‐analysis showed that the pH dependence of eDNA degradation was less pronounced in artificial water where microbial load was likely to be lower (e.g., tap and deionised water) compared to natural water (freshwater and seawater; Figure 1). Previous studies have suggested that eDNA decay rates might be lower in laboratory settings compared to field conditions owing to different microbial loads (Jo and Minamoto 2021; Lamb et al. 2022). This study newly proposed a possibility that the pH dependence of eDNA degradation may even vary between systems with different microbial loads.

This meta‐analysis also provides insights into the effects of other covariates, as well as their interactive effects with pH, on eDNA degradation. Warmer temperatures significantly increased eDNA decay rates without interacting with pH. Previous empirical studies and syntheses (Eichmiller et al. 2016; Lamb et al. 2022; Jo 2023) have also identified a role of temperature in eDNA decay rates, highlighting the significance of microbial metabolism and exonuclease activity on aqueous eDNA degradation irrespective of pH conditions (Strickler et al. 2015). In contrast, although eDNA decay rate was higher for longer DNA fragments (longer PCR amplicon) under relatively low pH conditions, degradation of longer DNA fragments was slower under relatively high pH conditions, as indicated by the interaction between pH and amplicon length. Frequent depurination and hydrolysis under acidic conditions may promote fragment length‐dependent eDNA degradation, whereas such chemical modifications are suppressed under alkaline conditions. There has been previous disagreement on when eDNA degradation depends on fragment lengths (Jo et al. 2017; Bylemans et al. 2018; Shogren et al. 2018; Jo et al. 2021; Saito and Doi 2021; Lamb et al. 2022), and the present meta‐analysis suggests that this discrepancy may be partly explained by a failure to account for the pH dependence of eDNA degradation. This finding highlights the importance of jointly considering the potential effects of multiple covariates on eDNA decay rates.

### 4.2 Possible Mechanisms Behind the Complexity of pH‐Dependent eDNA Degradation

**FIGURE 2:**
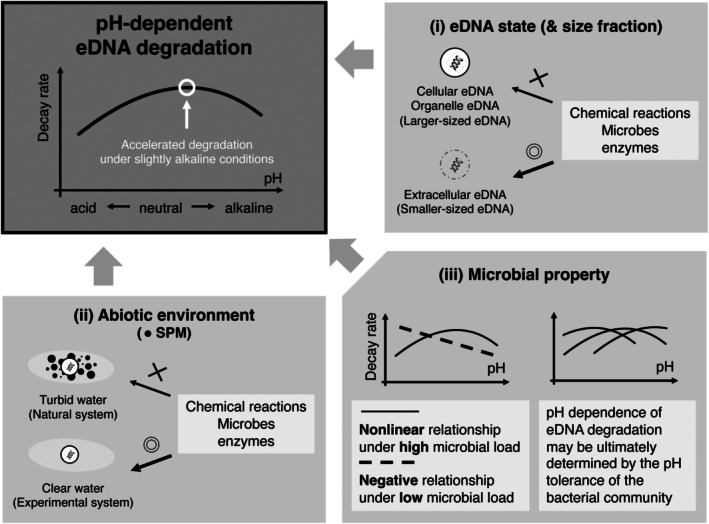
Schematic depiction showing the potential interactions between pH‐dependent eDNA degradation and additional factors. This study suggests a nonlinear relationship between eDNA decay rates and pH, with modelled eDNA decay rates peaking under slightly alkaline conditions; however, a pH‐dependent eDNA degradation should also consider the complex interplay between (i) eDNA state, (ii) abiotic environment, and (iii) microbial property. Regarding (i), cellular‐ and organelle‐derived eDNA in larger fractions should be more tolerant of microbial and enzymatic attacks compared to extracellular DNA‐derived eDNA in smaller fractions. The pH dependence of eDNA degradation may thus vary depending on its state in the water. Regarding (ii), eDNA may be more likely to adsorb onto suspended particle matter (SPM) in more turbid environments, which can protect the particle‐adsorbed eDNA from abiotic and biotic degradation. The pH dependence of eDNA degradation may thus vary depending on the amount of SPM in the water. Regarding (iii), the effects of pH on eDNA decay rates may depend on the microbial load and properties (e.g., pH tolerance).

Although this study revealed important findings regarding pH‐dependent degradation of macrobial eDNA, there was a substantial residual heterogeneity in the meta‐analysis. Figure 2 summarises some of the possible mechanisms behind the complexity of pH‐dependent eDNA degradation, each of which is discussed below.

#### 4.2.1 pH‐Dependent Variation in eDNA Degradation Related to eDNA States

As mentioned above, eDNA particles exist in various states in the water, and eDNA persistence is influenced by its cellular and molecular characteristics. Jo and Minamoto (2021) meta‐analysed previous studies and suggested that environmental factors (water temperature and quality) and eDNA states (size fraction and genetic region) could collectively influence the process of eDNA degradation. Some empirical studies have also demonstrated that the breakdown of larger eDNA particles, along with the inflow of eDNA from larger to smaller size fractions, could prolong the apparent persistence of the smaller‐sized eDNA (Jo et al. 2019; Brandão‐Dias, Hallack, et al. 2023), thereby complicating the pH dependence of eDNA degradation. Specifically, extracellular DNA‐derived eDNA in smaller fractions could be more susceptible to the influence of pH, including microbial and enzymatic processes, than cellular DNA‐derived eDNA in the larger fractions (Mauvisseau et al. 2022; Brandão‐Dias, Tank, et al. 2023). By using a larger pore size filter and selectively collecting larger eDNA particles, which increases the filtration efficiency and decreases the capture efficiency of microbes and contaminants (Turner et al. 2014; Jo, Takao, and Minamoto 2022; Allan et al. 2025), eDNA detection results may be less sensitive to degradation effects including pH at the sampling sites. Zhao et al. (2023) examined the relationship between pH and the decay rate of zebrafish ( *Danio rerio* ) eDNA using different filter pore sizes (1.2 to 5 and > 5 μm). Although they failed to detect an interactive effect of size fraction and pH on eDNA degradation, this was likely due to the very narrow pH range that was tested (pH: 7.62 to 8.34) and the fact that both size fractions contained predominantly cellular and/or organelle DNA and were too large to sufficiently collect dissolved DNA. Experiments conducted across broader ranges of both pH and filter pore sizes may help elucidate differences in pH‐dependent eDNA degradation between different eDNA states.

The characteristics of DNA fragments may also affect the complexity of pH‐dependent eDNA degradation regardless of PCR amplification length. DNA with a high GC content can be more chemically and thermally stable, and therefore more likely to experience slower degradation under low pH conditions, compared to AT‐rich sequences (Shchyolkina et al. 2000; Hu et al. 2022). The formation of secondary hairpin structures during PCR may also affect variability in DNA resistance to enzymatic degradation (Hirao et al. 1994).

#### 4.2.2 pH‐Dependent Variation in eDNA Degradation Associated With Suspended Particle Matter

In water, eDNA particles can be electrostatically adsorbed onto the surfaces of minerals and organic particles (e.g., dissolved organic matter), making the particle‐adsorbed eDNA more resistant to biotic and abiotic degradation than free, dissolved eDNA (Mauvisseau et al. 2022; Freeman et al. 2023). Eichmiller et al. (2016) demonstrated that decay rates of common carp ( *C. carpio* ) eDNA in 50‐mL experimental microcosms were negatively correlated with the amount of dissolved organic carbon (DOC), suggesting that binding DNA molecules to humic substances slows their degradation, potentially because nuclease activity is inhibited by chelation of metal ions (Wan et al. 2019). Particularly in acidic conditions, the stronger binding of negatively charged DNA molecules to the positively charged SPM surface may make particle‐adsorbed eDNA more protected from attacks by exonucleases (Romanowski et al. 1991; Allemand et al. 1997). Conversely, particle‐adsorbed eDNA could be physically removed from the water column by depositing onto substrates and may therefore not be detected (Brandão‐Dias, Tank, et al. 2023; Zhang et al. 2025), in which case the pH dependence of eDNA degradation may vary depending on the amount of suspended particle matter (SPM) in the water. In more turbid environments, eDNA may be more likely to adsorb onto SPM, and the effects of pH‐related abiotic (depurination and hydrolysis under acidic conditions) and biotic (enhanced microbial activity under neutral to moderately high pH levels) degradation may become obscured.

#### 4.2.3 pH‐Dependent Variation in eDNA Degradation Associated With Microbial Properties

The results of this study potentially reflect the effects of both the physicochemical instability of DNA molecules and the role of microbial and enzymatic activities, both of which might be mediated by particle adsorption effect as discussed above. The nonlinear and upward convex relationship between eDNA degradation and pH observed in the meta‐analysis should be clearer when the indirect effect of pH variation (i.e., enhanced bacterial and enzymatic activity under neutral to moderately high pH levels) is relatively large, as indicated by the interaction between pH and water types in the meta‐analysis. On the contrary, in conditions with low microbial load in the water, the contribution of the direct effect of pH variation (i.e., depurination and hydrolysis under acidic conditions) on eDNA degradation should be relatively large, potentially resulting in a negatively linear relationship between eDNA decay rates and pH (Strickler et al. 2015; Yang et al. 2025). Furthermore, in field‐collected (as opposed to experimental) water samples, the dominant bacterial community varies greatly depending on the pH conditions, with some bacterial communities adapted to extreme pH conditions such as acidic and highly alkaline environments (e.g., Bååth 1996; Quatrini and Johnson 2018; Amangeldina et al. 2024). Limited eDNA degradation at low pH levels in some case studies (Jo, Tsuri, and Yamanaka 2022; Kagzi et al. 2022) could be because the dominant bacterial community in the experimental tanks was not adapted to acidic conditions. Although most bacterial communities are thought to prefer neutral to slightly alkaline environments, the pH conditions under which eDNA degradation is maximised may ultimately be determined by the physiological properties of the microbial community that dominates at the study site.

#### 4.2.4 Other Factors

There may be some additional reasons why pH‐dependent eDNA degradation is complicated. Ion composition is a key factor determining pH in environments and may offer different effects on eDNA stability and adsorption dynamics. For example, some metal ions (e.g., Mg^2+^ and Ca^2+^) can bind to DNA similar to SPM; others like Fe^3+^ may promote DNA damage by catalysing oxidation reactions (Henle et al. 1996; Panteva et al. 2015). Moreover, in natural environments, light conditions can cause diel fluctuations in pH: during the day, CO_2_ absorption associated with photosynthesis increases pH while at night, CO_2_ release associated with respiration decreases pH. Although evidence that light intensity or type (e.g., ultraviolet‐A/B) significantly influences eDNA persistence under experimental conditions is scarce (e.g., Mächler et al. 2018), pH fluctuations associated with light conditions may indirectly influence nuclease activity and eDNA adsorption dynamics in the field (Fang et al. 2021).

### 4.3 Toward Expanding the Research Scope: Beyond Macrobial eDNA in the Water

This study limited its research focus to eDNA derived from macroorganisms (macrobial eDNA). However, macrobial eDNA constitutes only a small fraction of the total eDNA pool, with the vast majority originating from microbes such as bacteria (Turner et al. 2014). Macrobial eDNA consists primarily of extracellular and intracellular (but likely biologically inactive) DNA, whereas microbial eDNA also includes biologically active organisms. In particular, as microbes continue to metabolise and reproduce until they die, the dynamics of microbial eDNA from living organisms could be substantially different from those of extra‐organismal macrobial eDNA. Compared to macrobial eDNA that cannot increase without release from individuals, the dynamics of total microbial eDNA should not be monotonic. For example, living microbes physiologically adapt to pH changes in their environment (e.g., proton pumps, cell membrane transport, and gene expression regulation), while their death due to extreme pH changes may also cause temporary DNA release (Wan et al. 2020; Zhou et al. 2024). Therefore, the pH‐dependent degradation dynamics of microbial eDNA should be more complex than that of macrobial eDNA, suggesting the need to incorporate microbial ecological perspectives in future studies of microbial eDNA persistence.

This study also limited its research focus to aqueous eDNA, though eDNA is present in various environmental media, including soil, sediment, and air (Freeman et al. 2023; Sedlmayr and Schenekar 2024; Nousias et al. 2025). Because much of the eDNA in soil and sediment is extracellular DNA adsorbed to minerals and organic matter, it is more resistant to enzymatic degradation and environmental damage (including pH changes) than its aquatic counterpart (Nielsen et al. 2007; Freeman et al. 2023). However, the pH range and variability are generally greater in soil than in water (Mosley et al. 2024), which may diversify the mechanisms for adsorption, protection, and degradation of eDNA in soil systems. Moreover, resuspension of bottom substrates may increase SPM concentrations and alter microbial communities in water, potentially impacting the persistence dynamics of aqueous eDNA (e.g., degradation, adsorption, and retention) and its relationship with pH, as partly discussed above. In natural systems, water‐soil interactions occur continuously, and the differences in eDNA decay rate and its pH dependence could be assessed more directly by considering sediment and microbial loads as covariates. Unfortunately, due to the paucity of the studies in which these variables were quantified, the meta‐analysis only partly and indirectly addressed this point by accounting for water types (see above).

The recently identified potential of environmental RNA (eRNA) analysis for obtaining more recent species occurrence and physiological information (Jo et al. 2023; Hechler et al. 2025) may also require elucidating the relationship between eRNA degradation dynamics and environmental factors. In addition to the rapid degradation of eRNA compared to eDNA (Scriver et al. 2023), eRNA is considered unstable under alkaline conditions, unlike eDNA that is generally stable under such conditions, because the 3′, 5′‐phosphodiester linkage in RNA polymers is susceptible to alkaline hydrolysis (Li and Breaker 1999; Jo et al. 2023). Accordingly, eRNA degradation is likely to exhibit different dependency on pH conditions from eDNA degradation. Future studies could compare and integrate the degradation dynamics of these environmental nucleic acids (eNAs) under different environmental conditions.

## 5 Final Remarks

This study demonstrated an overall nonlinear relationship between eDNA decay rates and pH, with modelled eDNA decay rates peaking around pH 8, along with the complexity of interactions between pH and other covariates that affect eDNA degradation. Slightly alkaline conditions that are thought to facilitate DNA stabilisation and preservation are often found in natural environments including oceans (e.g., Collins et al. 2018). However, this study suggests that under these pH conditions eDNA degradation may instead be accelerated by the promotion of microbial and enzymatic activity, thereby highlighting a potential need to reconsider our understanding of pH‐dependent eDNA degradation when interpreting eDNA‐based biomonitoring results from sites with different water characteristics. This study also highlights the importance of measuring pH in parallel with eDNA sampling, which will enable a more accurate and reliable prediction of eDNA persistence time and decay rates under various environmental conditions (McCartin et al. 2022; Yates et al. 2021).

This study also identified some possible mechanisms that should be addressed in the future to achieve a more comprehensive understanding of the pH dependence of eDNA degradation. Although the meta‐analysis demonstrated a nonlinear relationship between eDNA decay rates and pH, the pH‐dependent eDNA degradation mechanism should ultimately be accounted for by the complex interplay among eDNA state, abiotic environment, and microbial properties, and likely cannot be predicted by pH alone (Figure 2). Of course, this conclusion is not limited to pH, but is also applicable to other environmental factors (e.g., temperature, UV and salinity). As discussed above, the risk of publication bias in the meta‐analysis was minimal, but there is little doubt that further studies are needed to take additional factors into account before we can completely understand the pH dependence of eDNA degradation. These efforts will contribute to improving the resolution of species detection via eDNA analysis, developing a more refined monitoring tool for aquatic biodiversity conservation and ecosystem management.

## Author Contributions

T.S.J. conceived the study, conducted a literature search and data analysis, and wrote the manuscript.

## Funding

This work was supported by the Grant‐in‐Aid for JSPS Research Fellows (grant numbers: JP22J00439 and JP22KJ3043).

## Disclosure

The proofreading of the manuscript was partly supported by Grammarly Pro (https://app.grammarly.com). After using this tool, I reviewed and edited the content as needed and took full responsibility for the content of the published article.

## Conflicts of Interest

The author declares no conflicts of interest.

## Supporting information


**Figure S1:** men70101‐sup‐0001‐Figures.docx.

**Table S1:** men70101‐sup‐0002‐Tables.xlsx.

## Data Availability

All the data supporting the findings of this study are provided in the main text and its Supporting Information.
