# Supplementary material for: pH‐Dependent Degradation of Macrobial Environmental DNA in Water
Source: Mol Ecol Resour. 2026 Jan 16;26(2):e70101. doi: 10.1111/1755-0998.70101 (PMC12809876; doi:10.1111/1755-0998.70101)
Supplement: Supplementary file 1 — Figure S1: men70101‐sup‐0001‐Figures.docx. [file MEN-26-e70101-s002.docx]

**Figure legends**

Figure S1. Relationships between eDNA decay rate constant [Yeo-Johnson-transformed; per hour] and (a) water temperature [°C] and (b) PCR amplicon length [bp]. The size of each plot represents the sample size (the number of water samples required to estimate the decay rate). A regression line is shown in a black using all the dataset in (a), while regression lines are shown in dark gray (pH < 8) and in light gray (pH ≧ 8).

Figure S2. Funnel plots in the meta-analyses using all dataset (left) and subset data (right).

Figure S1.

Figure S2.
